# Assessing precision and accuracy of manure volatile solids, total nitrogen, and total ammoniacal nitrogen analyses across 6 commercial laboratories

**DOI:** 10.3168/jdsc.2025-1007

**Published:** 2026-05-07

**Authors:** Haowen Hu, Jason P. Oliver, Puchun Niu, April B. Leytem, Kristan F. Reed

**Affiliations:** 1 Department of Animal Science, Cornell University, Ithaca, NY 14853; 2 Department of Animal Science, PRO-DAIRY Dairy Environmental Systems Program, Cornell University, Ithaca, NY 14853; 3 Department of Animal Sciences, Washington State University, Pullman, WA 99164

## Abstract

Manure nutrient analyses inform nutrient management plans and GHG emission inventories, yet the magnitude and sources of measurement variability remain under-studied for several key nutrients. To assess uncertainty in manure nutrient measurements, we quantified intra- and interlaboratory variation and analytical accuracy for volatile solids (VS), total nitrogen (TN), and total ammoniacal nitrogen (TAN) across 6 US laboratories certified by the Manure Analysis Proficiency Program. Fresh gutter-collected slurry subsamples (250 g) from a freestall barn received either an equal amount of water (control), one of 2 spike levels of glucose (for VS), or one of 2 spike levels of NH_4_Cl (for TN and TAN). Repeatability (V_r_ = repeatability value; SD_r_ = within-laboratory SD; RSD_r_ = relative SD of repeatability) and leave-one-lab-out reproducibility (V_R_ = reproducibility value; SD_R_ = between-laboratory SD; RSD_R_ = relative SD of reproducibility) were calculated to evaluate precision. Spike recovery was used to assess accuracy. Repeatability values ranged from 0.30% to 3.49% for VS, 16.2 to 932 mg/kg for TN, and 28.0 to 1.65 × 10^3^ mg/kg for TAN, whereas values for leave-one-lab-out V_R_ ranged from 2.56% to 6.08%, 405 to 683 mg/kg, and 363 to 593 mg/kg, respectively. Variability among laboratories was consistently higher than variability within laboratories but differed by nutrient: overall RSD_R_ averaged 23.3% for VS, 6.32% for TN, and 15.6% for TAN. Mean recovery rates for nutrient-by-spike-level combinations across laboratories ranged from 64.1% to 118.7%, with all but 5 mean values falling within the literature-recommended ranges (70% to 110% for VS and 85% to 115% for TN/TAN). Results confirm glucose and NH_4_Cl as effective positive control spikes and highlight the impact of analytical method on VS and TN agreement across laboratories. These findings provide metrics of uncertainty in manure nutrient data that should be considered when measurements are used in research, modeling, and on-farm decision-making.

## Highlights

• Laboratories were more consistent within themselves than with each other.
• Precision varied most for VS and least for TN across laboratories.
• Accuracy was generally strong, with most spike recoveries in target ranges.
• Glucose and NH4Cl solutions confirmed as reliable positive control spikes.

## Graphical Abstract

**Figure:**
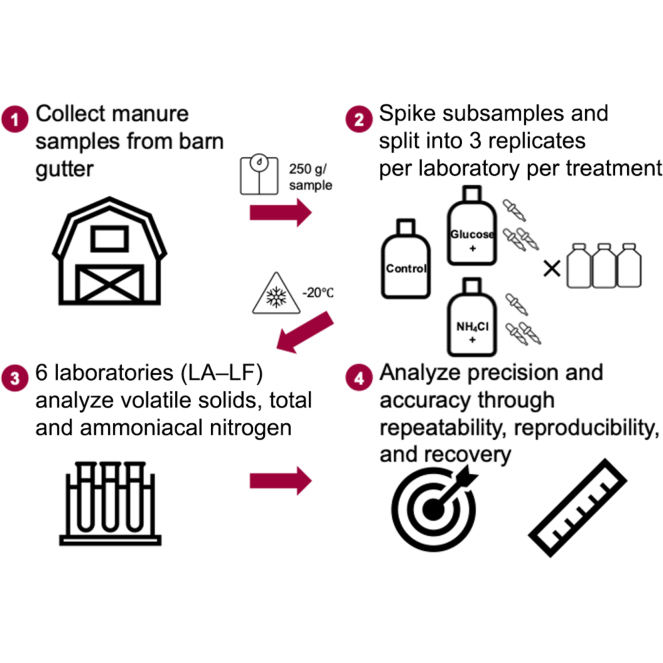
**Summary:**Barn-gutter manure samples were collected, homogenized, and subdivided into subsamples that received either water (control), glucose spikes for volatile solids (VS), or NH_4_Cl spikes for total and ammoniacal nitrogen (TN and TAN) at 2 levels each in replicates of 3. Subsamples were frozen before shipment to 6 proficiencycertified laboratories (LA-LF), which measured VS, TN, and TAN using their standard methods. Analytical performance was evaluated through repeatability (within-laboratory precision), reproducibility (betweenlaboratory precision), and spike recovery (accuracy). Within-laboratory precision was higher than betweenlaboratory precision and differed by nutrient. Variability was highest for VS and lowest for TN. Most spike recoveries were within the recommended ranges. Our study provides uncertainty metrics for manure nutrient analyses and underscores the need for strong quality control in commercial laboratories to support research, modeling, and on-farm decision-making.

Livestock manure, composed of feces, urine, and soiled bedding, is a valuable source of nutrients, benefiting soil health and crop growth (Sawatdeenarunat et al., 2016; Rayne and Aula, 2020). Between 1860 and 2017, total manure nitrogen production in the contiguous United States increased from 1.4 Tg/yr to 7.4 Tg/yr (Bian et al., 2021). However, the increase in manure generation and use can lead to environmental challenges such as GHG emissions and ecosystem eutrophication (Varma et al., 2021). Globally, manure management contributes approximately 10% of agricultural GHG emissions (Owen and Silver, 2015). In the United States, manure management contributes to 13.3% of total GHG from agriculture (EPA, 2022). The primary GHG emitted from manure are methane (CH_4_) and nitrous oxide (N_2_O). Methane emissions result from and are predicted by microbial anaerobic degradation of organic matter, also known as volatile solids (**VS**; Rivera and Chará, 2021). Likewise, N_2_O emissions arise through the microbial processes of nitrification and denitrification (Rivera and Chará, 2021). Ammonia (NH_3_) gas is also produced during manure management, and although it is not a GHG, it serves as a precursor for N_2_O and represents a source of nutrient loss (Hristov et al., 2026). Ammonia originates from the volatilization of total ammoniacal nitrogen (**TAN**), which is formed from the breakdown of urea and other nitrogenous compounds during various stages of manure management, including animal housing, storage, land application, and grazing (Webb et al., 2005; Behera et al., 2013). Manure VS, total nitrogen (**TN**), and TAN composition values are used to inform nutrient management plans and predict environmental losses and impacts. Therefore, accurately quantifying manure VS, TN, and TAN content is critical for both nutrient management and environment sustainability efforts.

Average manure nutrient contents are available in the literature, but manure nutrients vary in response to many factors including diet, animal type and genetics, bedding, handling, and storage (Wilson et al., 2022). A widely accepted and dependable method for assessing the nutrient content of manure is by collecting a representative sample and submitting it to a laboratory for testing (Wilson et al., 2022). A national laboratory proficiency testing program for manure, the Manure Analysis Proficiency (**MAP**) Program, was developed by the Minnesota Department of Agriculture in 1996 to ensure minimum standards for accuracy and precision in manure nutrient analysis (Floren et al., 2006). Since then, some assessment of intra- and interlaboratory variation of the analysis of manure nutrients have been presented in the literature. Sanford et al. (2020) sent 6 replicates of 4 manure samples, 2 from dairy and 2 from swine, to 8 MAP-certified laboratories. They reported high precision and accuracy in TN measurements across laboratories, although analysis of TAN was notably less consistent for both accuracy and precision. Importantly, VS were not included in their study. Similarly, Wilson et al. (2022) assessed the intralaboratory repeatability and interlaboratory reproducibility of TS measurements from various manure samples. They reported interlaboratory SD between 0.27% and 1.16% for manure samples with average TS contents ranging from 3.15% to 68.4%. However, like the Sanford et al. (2020) study, no results were provided for VS. Thus, this project aims to (1) assess analytical precision by quantifying both the differences between (inter-) laboratories and the consistency within individual (intra-) laboratories for selected MAP-certified laboratories across the United States when analyzing manure nutrients such as VS, TN, and TAN; and (2) assess analytical accuracy for these nutrients by determining the recovery rates of known nutrient spikes included as positive controls.

Dairy manure samples were collected at the Cornell Dairy Research and Education Center (Harford, NY) in April 2025. At this farm, manure is managed by scraping each pen separately into a central gutter while cows are being milked in the parlor. To obtain a representative sample from a single pen, manure was collected into a 38-L (10-gallon) bucket immediately after scraping using a 12.7-cm (5-inch) diameter scoop (0.95-L [0.25-gallon] capacity) attached to a telescoping handle, and a shovel for manure surrounding the gutter. Manure was mixed thoroughly using a drill-powered mixing paddle to ensure uniformity while 250-g subsamples were taken for laboratory analysis.

Known quantities of positive control spikes were added to the subsamples to assess the accuracy of laboratory assays. We selected a glucose solution (455 g/L) and an ammonium chloride (NH_4_Cl) solution (250 g/L) as the positive control spikes for VS and TN/TAN, respectively. Each positive control spike was assigned at 2 dosage levels, low (level 1) and high (level 2). The high dosage was calculated to achieve a total concentration in the spiked sample that reflected the upper bound of reported concentration values by ManureDB (http://manuredb.umn.edu/), a manure database hosted by the University of Minnesota, accounting for an assumed average initial concentration in the sample. The low dosage was similarly calculated to achieve an intermediate total concentration. Specific spike amounts are presented in Table 1. To maintain consistency in total sample weight and TS content, an equivalent amount of water was added to control manure subsamples. This resulted in a total of 5 treatments, including the control group. Three replicates per treatment were prepared for each selected laboratory (6 total, described in the following paragraph). Subsamples were randomly assigned within treatment group, resulting in a total of 90 subsamples (5 treatments × 3 replicates/treatment × 6 laboratories). Before and after addition of spikes, we measured the pH of 3 replicates per treatment group using a calibrated pH electrode (Orion 4-Star, Thermo Scientific, Waltham, MA). Subsamples were then frozen at −20°C before shipment to laboratories for analysis.

**Table 1:** Summary of subsample weight, spike additions, and pH measurements^1^

Subsample property	Level 1 (mean ± SD)	Level 2 (mean ± SD)
Spike amount		
Glucose added, g	16.4 ± 0.167	33.1 ± 0.291
NH_4_Cl added, g	4.04 ± 0.094	15.5 ± 0.094
Additional VS,^2^ g	6.79 ± 0.067	13.7 ± 0.122
Additional TN/TAN,^2^ g	0.253 ± 5.6 × 10^3^	0.972 ± 4.6 × 10^3^
pH measurement		
Glucose-treated	8.74 ± 0.075	8.72 ± 0.025
NH_4_Cl-treated	8.61 ± 0.072	8.34 ± 0.055

1 Spike amount values represent means across laboratories; pH values are means of 3 randomly selected subsamples per treatment. Level 1 and level 2 refer to low and high spike dosages, respectively. Manure subsample weight was 250.0 ± 2.45 g before addition of water or spikes.

2 “Additional VS” and “additional TN/TAN” denote the mass of volatile solids (VS), total nitrogen (TN), and total ammoniacal nitrogen (TAN) contributed by the spike solutions.

Following the approach of Sanford et al. (2020), we selected 6 laboratories from the 2025 MAP-certified laboratories (https://www2.mda.state.mn.us/webapp/lis/manurelabs.jsp) and anonymized them as **LA** through **LF**. Manure samples were analyzed for VS, TN, and TAN. Table 2 summarizes each laboratory's methods and, if applicable, the minimum manure quantity required per sample. For data analysis, VS, TN, and TAN concentrations were expressed on a wet basis, with TS and VS reported as percentages and TN and TAN as milligrams per kilogram, following Wilson et al. (2022). Unless otherwise stated, values are expressed as mean ± SD. Analytical methods for VS, TN, and TAN used by the enrolled laboratories are described in the following paragraph.

**Table 2:** Comparison of analytical methods and sample quantity requirements across selected laboratories^1^

Laboratory	Methodology			Minimum amount
	VS	TN	TAN	
LA	Loss on ignition (ash @ 550°C)	Total Kjeldahl	NA	250 g
LB	Loss on ignition	Total Kjeldahl	KCl extraction	250 mL
LC	Loss on ignition (ash @ 600°C)	Total Kjeldahl or combustion	Diffusion analysis	237 mL (8 oz)
LD	Loss on ignition (ash @ 550°C)	Combustion	Titration	NA
LE	Loss on ignition (ash @ 550°C)	Combustion	KCl extraction	NA
LF	Loss on ignition	Total Kjeldahl or combustion	Flow injection analysis or titration	150 g

1 VS = volatile solids; TN = total nitrogen; TAN = total ammoniacal nitrogen: NA = not available.

Volatile solids were determined by loss on ignition, calculated as the difference between TS (after drying at 103°C to 105°C) and fixed solids (after further heating at 550°C ± 50°C; Wilson et al., 2022). Total nitrogen was measured by either combustion, in which manure is oxidized in a high-temperature furnace (>900°C) in the presence of oxygen, or by the Kjeldahl method, where samples are digested with concentrated sulfuric acid to convert organically bound nitrogen into ammonium (Wilson et al., 2022). Total ammoniacal nitrogen was quantified by extracting ammonium nitrogen with acidified 1.0 *M* KCl or from a total Kjeldahl nitrogen digest, followed by one of 3 analysis methods: distillation/titration, flow injection, or diffusion-conductivity (Wilson et al., 2022).

Intralaboratory repeatability and leave-one-lab-out interlaboratory reproducibility were calculated following Lynch (1998) to assess precision. Analyses used laboratory-reported concentrations of VS, TN, and TAN from control groups, corrected for added water. Precision metrics are reported as the repeatability value (**V_r_**), its SD (**SD_r_**), and its relative SD (**RSD_r_**), as well as the reproducibility value (**V_R_**), its SD (**SD_R_**), and its relative SD (**RSD_R_**). The SD_r_ and SD_R_ are calculated using *n*− 1 degrees of freedom, where *n* is the number of observations used in each SD calculation. The V_r_ and V_R_ values are calculated by multiplying the SD by a factor of 2.8, which is derived from the 95% prediction interval for the difference between 2 measurements. The RSD_r_ and RSD_R_, also known as the coefficients of variation, are calculated as the SD as a percentage of the mean analysis result (Lynch, 1998). To compute the leave-one-lab-out V_R_ for each laboratory *J* (*R*_(−_*_J_*_)_, *J* = LA through LF), we excluded observations from laboratory *J* and then calculated the SD of the means from the remaining laboratories as follows:

[1] $$SD_{R\left(-J\right)}=\sqrt{\frac{1}{\left(n-2\right)}\sum_{j\neq J}{\left({\bar{X}}_{j}-{\bar{\bar{X}}}_{\left(-J\right)}\right)}^{2}},$$

where ${\bar{X}}_{j}$is the mean from laboratory *j*, *J* is the laboratory excluded in the leave-one-lab-out calculation, and ${\bar{\bar{X}}}_{\left(-J\right)}$ is the mean of all ${\bar{X}}_{j}$ excluding laboratory *J*:

[2] $${\bar{\bar{X}}}_{\left(-J\right)}=\frac{1}{\left(n-1\right)}\sum_{j\neq J}{\bar{X}}_{j}.$$

The leave-one-lab-out V_R_ was then computed as

[3] V_R(−_*_J_*_)_ = 2.8 × SD_R(−_*_J_*_)_.

The leave-one-lab-out approach quantifies reproducibility based on the variability among the other laboratories; a lower V_R(−_*_J_*_)_ value indicates a greater contribution of laboratory *J* to the overall interlaboratory variation. In addition, we reported the overall RSD_R_, calculated using the mean and SD across all laboratories.

To test for differences between laboratories, we applied a linear mixed model with laboratory as a random effect to the control group measurements using Python (v3.13, Python Software Foundation). Residuals were tested for normality using the Shapiro–Wilk test (Shapiro and Wilk, 1965), with a *P*-value greater than 0.05 indicating no evidence against the normality assumption. When residuals were normally distributed, differences among laboratory means were evaluated using one-way ANOVA (Ross and Willson, 2017), followed by Tukey's honestly significant difference (**HSD**) test (Chmiel et al., 2022) for pairwise comparisons. When normality was not met, a Kruskal–Wallis test (McKight and Najab, 2010) was applied, and significant results were further examined using Dunn's test (Dunn, 1964) with Bonferroni correction. A significance level of 0.05 was applied throughout.

Accuracy is estimated as the recovery rate of a positive control spike present in known concentrations (Thompson et al., 1999). Specifically, it is calculated as the ratio of reported concentration of a positive control spike to that expected to be present, expressed as a percentage, as shown in Equation 4:

[4] $$Recovery_{j}=\frac{c_{reported,\;i,\;j}}{\frac{w_{manure,\;i,\;j}\times \frac{c_{j}}{100}+w_{spike,\;i,j}\;}{w_{tot,\;\;i,j}}\times 100}\times 100,$$

where *Recovery_j_* is the recovery rate (%) of VS, TN, or TAN at each spike level by laboratory *j* (*j*= LA through LF); *c_reported_*_,_*_i_*_,_*_j_* is the reported concentration (%) of VS, TN, or TAN of subsample *i* (*i*= 1 to 3 for each level of each positive control spike) measured by laboratory *j*; *c_j_*is the average concentration (%) of VS, TN, or TAN of the replicate samples from the control group of laboratory *j* after correction for additional water; *w_manure_*_,_*_i_*_,_*_j_* refers to the weight (g) of the *i*th manure subsample received by laboratory *j* and *w_tot_*_,_*_i_*_,_*_j_* indicates the total weight (g) of the *i*th manure subsample with the addition of a positive control spike; and *w_spike_*_,_*_i_*_,_*_j_* represents the amount (g) of VS, TN, or TAN added to the *i*th manure subsample received by laboratory *j*, calculated based on the added amount of positive control spike solutions, their known density, and molecular weight conversions (100% of glucose = VS, 26% of NH_4_Cl = TN/TAN). The average recovery rate for a given nutrient (VS, TN, or TAN) at a positive spike level (1 or 2) was considered acceptable when it ranged from 70% to 110% for VS and from 85 to 115% for TN/TAN (Wilson et al., 2022).

Manure subsample weights and the amounts of glucose and NH_4_Cl spikes added for both levels were consistent across laboratories (Table 1). Manure pH before spiking averaged 8.74 ±0.015, consistent with the 7.46 to 8.81 range reported by Amador et al. (2019). The pH of control samples remained stable after water addition (8.78 ± 0.055), whereas glucose-treated samples showed minimal change. The NH_4_Cl-treated samples exhibited a numerical drop in pH, likely due to the acidic nature of the NH_4_Cl solution (Table 1).

Our statistical analysis indicated that VS and TN measurements met the normality assumption (*P* > 0.05), but TAN did not (*P* < 0.001), suggesting that laboratory differences for VS and TN can be interpreted using parametric comparisons, whereas TAN requires nonparametric evaluation. Repeatability and reproducibility metrics are summarized in Table 3. For corrected VS concentration, LC exhibited the poorest repeatability (SD_r_: 1.25%, RSD_r_: 10.4%). Furthermore, LC largely affected overall interlaboratory variation, as its exclusion resulted in the lowest SD_R_ (0.914%) and RSD_R_ (10.9%) among the remaining laboratories. This was supported by Tukey HSD test results, which showed LC's mean corrected VS concentration to be significantly higher than other laboratories (*P* < 0.001). For corrected TN concentration, repeatability was also the poorest with LC (SD_r_: 333 mg/kg, RSD_r_: 9.17%). Leave-one-lab-out reproducibility for corrected TN concentration, however, was the best when LD was excluded (SD_R_: 145 mg/kg, RSD_R_: 4.10%). The mean corrected TN concentration from LD was significantly lower than LA, LB, LC, and LF, with *P*-values of 0.030, 0.009, 0.040, and 0.003, respectively. For corrected TAN concentration, repeatability was the poorest for LB (SD_r_: 588 mg/kg, RSD_r_: 41.9%), whereas leave-one-lab-out reproducibility for corrected TAN concentration was the best when LF was excluded (SD_R_: 130 mg/kg, RSD_R_: 10.6%). Even though the Kruskal–Wallis test indicated significant differences in corrected TAN concentrations among laboratories (*P* = 0.044), Dunn's test did not identify any specific pairwise differences (*P* > 0.05).

**Table 3:** Precision (repeatability and reproducibility) and spike recovery accuracy of manure nutrient analyses across 6 laboratories

Laboratory	Nutrient^1^	Corrected mean,^2^ mg/kg	Intralaboratory repeatability standard deviation (SD_r_), mg/kg	Intralaboratory repeatability value (V_r_), mg/kg	Relative repeatability standard deviation (RSD_r_), %	Interlaboratory reproducibility standard deviation (SD_R_), mg/kg	Interlaboratory reproducibility value (V_R_), mg/kg	Relative reproducibility standard deviation (RSD_R_), %	Level 1 positive spike recovery, %	Level 2 positive spike recovery, %
LA	TS^3^	21.0	0.153	0.429	0.730	2.81	7.88	14.0	NA	NA
	VS^3^	6.57^a^	0.290	0.812	4.41	1.94	5.43	23.1	97.2 ± 5.4	86.2 ± 2.8
	TN	3.66 × 10^3^^b^	152	425	4.15	240	672	6.80	100.2 ± 1.8	99.8 ± 2.6
	TAN	1.25 × 10^3^^a^	115	323	9.23	212	593	17.4	98.5 ± 3.6	93.5 ± 2.9
LB	TS^3^	22.1	0.498	1.39	2.26	2.67	7.46	13.4	NA	NA
	VS^3^	6.99^a^	0.180	0.505	2.58	2.04	5.72	24.3	99.9 ± 3.7	98.8 ± 5.5
	TN	3.74 × 10^3^^b^	5.77	16.2	0.154	221	619	6.26	103.8 ± 4.8	101.0 ± 0.4
	TAN	1.40 × 10^3^^a^	588	1.65 × 10^3^	41.9	187	523	15.3	76.8 ± 2.6	82.8 ± 7.6
LC	TS^3^	22.0	1.09	3.06	4.97	2.68	7.49	13.4	NA	NA
	VS^3^	12.0^c^	1.25	3.49	10.4	0.914	2.56	10.9	110.0 ± 11.7	102.6 ± 4.1
	TN	3.63 × 10^3^^b^	333	932	9.17	244	683	6.90	92.4 ± 4.8	93.9 ± 2.6
	TAN	1.07 × 10^3^^a^	10.0	28.0	0.935	197	551	16.1	92.5 ± 5.1	96.1 ± 3.6
LD	TS^3^	15.3	3.78	10.6	24.7	0.813	2.28	3.82	NA	NA
	VS^3^	8.89^b^	0.397	1.11	4.46	2.16	6.05	25.8	103.5 ± 21.6	92.9 ± 2.5
	TN	3.16 × 10^3^^a^	120	336	3.80	145	405	4.10	94.1 ± 1.6	89.9 ± 10.9
	TAN	1.35 × 10^3^^a^	110	308	8.14	199	558	16.4	118.7 ± 44.6	64.1 ± 38.9
LE	TS^3^	20.1	0.584	1.64	2.91	2.83	7.94	13.9	NA	NA
	VS^3^	8.05^ab^	0.430	1.21	5.35	2.17	6.08	25.9	90.7 ± 2.0	87.6 ± 1.4
	TN	3.36 × 10^3^^a^	34.6	97.0	1.03	231	648	6.55	96.2 ± 7.5	88.4 ± 1.3
	TAN	1.32 × 10^3^^a^	34.6	97.0	2.62	205	574	16.8	98.1 ± 4.4	100.7 ± 3.6
LF	TS^3^	21.3	1.14	3.20	5.36	2.78	7.79	13.8	NA	NA
	VS^3^	7.82^ab^	0.108	0.303	1.38	2.16	6.04	25.7	111.7 ± 22.3	97.7 ± 1.6
	TN	3.64 × 10^3^^b^	104	292	2.87	243	679	6.87	100.5 ± 4.9	98.7 ± 3.4
	TAN	910^a^	70.0	196	7.69	130	363	10.6	103.0 ± 2.1	94.9 ± 1.5

a,b Within each nutrient, means with different superscript letters differ significantly (*P* < 0.05, post hoc multiple comparison test). Means sharing at least one letter are not significantly different from groups labeled with either of those letters.

1 VS = volatile solids; TN = total nitrogen; TAN = total ammoniacal nitrogen.

2 Means were adjusted by accounting for the additional water added to control group samples.

3 TS and VS mean, SD_r_, V_r_, SD_R_, and V_R_ values are given in % rather than mg/kg.

Interlaboratory variation also showed differences depending on the nutrients. Higher interlaboratory variation was observed for corrected TAN concentration (overall RSD_R_: 15.6%) compared with corrected TN concentration (overall RSD_R_: 6.32%). This is consistent with the general observation that analytes at lower concentrations tend to have greater RSD (Lynch, 1998). However, interlaboratory variation of corrected TAN concentration was lower than that of corrected VS concentration (overall RSD_R_: 23.3%), despite TAN's lower average concentration. This anomaly for VS could be attributed to the much larger VS concentration measured by LC (Table 3).

Intra- and interlaboratory variation can arise from multiple sources. Intralaboratory variation may result from human error, sample handling, instrument and method selection, and data processing and reporting (Sanford et al., 2020; Wilson et al., 2022). For example, LB and LD reported TN values to only 2 decimal places, whereas the other 4 laboratories used 3, potentially affecting repeatability results. Sources of variation across laboratories include differences in analytical techniques, reporting formats, and unit-conversion choices (e.g., use of different density factors; Sanford et al., 2020; Wilson et al., 2022). For TN analysis, LA and LB used the Kjeldahl method, LD and LE used combustion, and LC and LF listed both methods without specifying which was applied. To determine VS, LC conducted loss on ignition at 600°C, whereas LA, LD, and LE used 550°C, potentially contributing to its greater interlaboratory variation.

Across all laboratories, V_r_ ranged from 0.303% to 3.49% for VS, 16.2 to 932 mg/kg for TN, and 28.0 to 1.65 × 10^3^ mg/kg for TAN. Leave-one-lab-out V_R_ ranged from 2.56% to 6.08% for VS, 405 to 683 mg/kg for TN, and 363 to 593 mg/kg for TAN. Except for VS and TN from LC and TAN from LB, intralaboratory variation was smaller than interlaboratory variation, consistent with Wilson et al. (2022). Comparison with literature revealed generally comparable V_r_ and V_R_ in the present study. Because repeatability data for VS are limited, TS were used as a proxy. The V_r_ (0.303 to 3.49%) and V_R_ (2.56%, leaving LC out) for VS were higher than those reported by Wilson et al. (2022; V_r_: 0.057%; V_R_: 0.93%) for manure with similar TS content. Likewise, TS V_r_ (0.429 to 10.6%) and V_R_ (2.28%, leaving LD out; Table 3) in our data were also greater than values in Wilson et al. (2022), which may reflect inadequate homogenization, subsampling, or weighing practices in some laboratories. After excluding measurements from LD, the V_R_ for TN (405 mg/kg) was better than the values reported for the Kjeldahl method (540 mg/kg) and the combustion method (1,780 mg/kg) by Wilson et al. (2022). For laboratories using Kjeldahl (LA and LB), LB (V_r_ = 16.2 mg/kg) showed better repeatability than the reference (54 mg/kg). Laboratories using combustion (336 mg/kg for LD and 97.0 mg/kg for LE) also outperformed the reference V_r_ (370 mg/kg). After removing TAN data from LF, V_R_ (363 mg/kg) was comparable to Wilson et al. (2022), who reported 1,098, 342, and 431 mg/kg for titration, spectrophotometric, and diffusion analyses, respectively. Laboratory C (28.0 mg/kg) outperformed the reported V_r_ (87.8 mg/kg) for diffusion analysis of TAN, whereas repeatability in laboratories using titration analysis (V_r_ = 308 mg/kg for LD and 196 mg/kg for LF) was poorer than the reported 64.5 mg/kg (Wilson et al., 2022). These differences likely reflect variations in sample type (storage vs. housing manure), homogenization method (food processor by Wilson et al., 2022 vs. drill mixer in the present study), and additional water applied to control samples.

Mean corrected concentrations for the control groups, after excluding laboratories with the lowest leave-one-lab-out V_R_, were 7.66% ± 0.912% for VS (excluding LC), 3.61 ± 0.15 × 10^3^ mg/kg for TN (excluding LD), and 1.28 ± 0.13 × 10^3^ mg/kg for TAN (excluding LF). These measurements are comparable to publicly reported values. Oliver et al. (2018) observed a VS concentration of 7.8% for blend-pit manure slurry collected at one sand-bedded dairy farm in the Northeastern United States. Similarly, Sokolov et al. (2019) reported VS, TN, and TAN concentrations of 8.6%, 4.5 × 10^3^ mg/kg, and 1.9 × 10^3^ mg/kg, respectively, for raw dairy manure collected from farm storage at a tiestall dairy facility with sand bedding in Canada. Data from ManureDB (http://manuredb.umn.edu/) reveal a median TN concentration of 4.0 × 10^3^ mg/kg and TAN of 1.0 × 10^3^ mg/kg, respectively, for nonliquid dairy manure, as classified by ManureDB, in the state of New York between 2021 and 2024.

Laboratory accuracy, reflected by recovery rates, ranged from 64.1% ± 38.9% to 118.7% ± 44.6% and varied by nutrient (Table 3). Laboratory B consistently demonstrated high accuracy for VS and TN measurements across both spike levels, with recovery rates ranging from 98.8% ± 5.5% to 103.8 ± 4.8% (Table 3). After removal of a visual outlier, LF also had relatively high accuracy in VS analysis with recovery of 97.5% ± 1.8% of the glucose in the level 1 group. However, in practical scenarios where a single sample is submitted for analysis, outliers would not be detected. For TN, LA and LF also exhibited strong accuracy, between 98.7% ± 3.4% and 100.5% ± 4.9%. Laboratories A, E, and F provided reliable TAN recoveries, ranging from 93.5% ± 2.9% to 103.0% ± 2.1%. In contrast, despite its high accuracy in VS and TN, the TAN analysis results of LB resulted in low recovery rates of 76.8% ± 2.6% for NH_4_Cl-N in level 1 and 82.8% ± 7.6% for NH_4_Cl-N in level 2. This lower accuracy was likely connected to substantial intralaboratory variation seen for the TAN analysis in this laboratory. Even after removing a visual outlier, LB's TAN recovery rates improved only to 89.2% ± 2.7% for NH_4_Cl-N in level 1 and 88.4% ± 8.3% for NH_4_Cl-N in level 2, still below the accuracy observed for LA, LE, and LF. In summary, except for 1 VS and 4 TAN mean recovery values, all nutrient-by-spike-level mean recoveries were within the recovery ranges (70% to 110% for VS and 85% to 115% for TN/TAN) recommended by Wilson et al. (2022).

Positive control spikes are essential in in vitro assays to confirm that the measurement system is functioning properly and to ensure results are robust and reproducible across different studies, laboratories, and over time (Petersen et al., 2021). Previous research identified coffee filters and microcrystalline cellulose as reliable positive control spikes for VS (Koch et al., 2020). Our results further demonstrate that glucose solution is also a suitable candidate for this purpose, as evidenced by consistent VS recovery rates across laboratories and stable pH following glucose addition. Ammonium salts, such as NH_4_Cl and ammonium sulfate, are types of nitrogen fertilizers (Nadarajan and Sukumaran, 2021). In this study, we used NH_4_Cl solution as the positive control spike for both TN and TAN. Although NH_3_ volatilization can occur, particularly when pH exceeds 7 (Rochette et al., 2013), no systematic bias was observed in the results from the positive controls. Additionally, the short turnaround time between adding the NH_4_Cl spike and freezing the samples likely minimized any loss. These findings support the use of ammonium salts like NH_4_Cl as suitable positive control spikes for TN and TAN. Furthermore, we observed that adding NH_4_Cl led to elevated VS measurements across laboratories, with varying degrees of increase. This is expected, as the loss on ignition method calculates VS as the difference between TS and fixed solids. Volatilization of NH_4_Cl contributes to this loss, inflating VS values, even though NH_4_Cl is inorganic. Some laboratory reports may refer to VS as organic matter, despite this limitation. Wilson et al. (2022) also stated that the loss on ignition method cannot reliably distinguish between organic and inorganic components, as some mineral salts also volatilize. Consequently, in our analysis of positive controls, we used NH_4_Cl groups for TN and TAN, and glucose groups for VS.

In conclusion, this study successfully quantified the magnitude of measurement variation of manure VS, TN, and TAN, fulfilling our primary objective. It also highlights the importance of generating high-quality, reliable data to support downstream research, modeling, and decision-making in livestock manure management (Sharma et al., 2026). Given the similarities between manure and soil, these findings may also serve as a reminder for soil-related studies to implement rigorous data quality control. Future evaluations of variation may include results from additional analytical methods, such as near infrared reflectance spectroscopy for TN and ion-selective electrode analysis for TAN.
